# Supplementary material for: Observing one-divalent-metal-ion-dependent and histidine-promoted His-Me family I-PpoI nuclease catalysis in crystallo
Source: eLife. 2024 Aug 14;13:RP99960. doi: 10.7554/eLife.99960 (PMC11325842; doi:10.7554/eLife.99960)
Supplement: Supplementary file 1. [file elife-99960-supp1.docx]

**Supplementary File 1. Crystal Diffraction and refinement data.**

(**A**) I-PpoI-DNA complex reaction with 500 µM Mg^2+^ at pH 7.0.

|  | PRS (pH 7.0) | 10 s | 20 s | 40 s |
| --- | --- | --- | --- | --- |
| **PDB Code** | 8VMO | 8VMP | 8VMQ | 8VMR |
| **Data collection** | |  |  |  |
| Wavelength (Å) | 0.9786 | 0.9787 | 0.9787 | 0.9787 |
| Space group | *P3_1_21* | *P3_1_21* | *P3_1_21* | *P3_1_21* |
| Cell dimensions | |  |  |  |
| *a, b, c* (Å) | 114.083 | 113.58 | 113.58 | 113.58 |
|  | 114.083 | 113.58 | 113.58 | 113.58 |
|  | 88.123 | 87.99 | 87.99 | 87.99 |
| *α, β, γ* (˚) | 90, 90, 120 | 90, 90, 120 | 90, 90, 120 | 90, 90, 120 |
| Resolution (Å)^1^ | 49.4 - 1.68 | 37.18 - 1.45 | 37.18 - 1.48 | 37.18 - 1.5 |
|  | (1.74 - 1.68) | (1.502 - 1.45) | (1.533 - 1.48) | (1.554 - 1.5) |
| R_sym_ or R_merge_^1^ | 0.09259 (0.9459) | 0.08503 (0.7265) | 0.07876 (0.9328) | 0.08952 (1.033) |
| *I*/σ*I*^1^ | 15.60 (2.31) | 13.95 (2.24) | 16.47 (2.20) | 14.32 (1.95) |
| CC^1/2 1^ | 0.999 (0.838) | 0.998 (0.836) | 0.999 (0.803) | 0.999 (0.814) |
| Completeness (%) | 99.99 (100.00) | 99.50 (98.81) | 99.90 (99.21) | 99.97 (99.86) |
| Redundancy^1^ | 11.0 (10.9) | 9.0 (8.3) | 10.9 (10.1) | 10.9 (10.2) |
| No. unique reflections^1^ | 75539 (7463) | 115178 (11274) | 108855 (10719) | 104681 (10370) |
| **Refinement** | |  |  |  |
| Me_1_ | 1.0 Na^+^ | 1.0 Na^+^ | 0.1 Mg^2+^ | 0.2 Mg^2+^ |
|  |  |  | 0.9 Na^+^ | 0.8 Na^+^ |
| Me_2_ | 1.0 Na^+^ | 1.0 Na^+^ | 1.0 Na^+^ | 1.0 Na^+^ |
| Product_1_ | - | - | 0.1 | 0.2 |
| Product_2_ | - | - | - | - |
| B-factors |  |  |  |  |
| Me_1_/Lig_1_ ^2^ | 20.7/22.0 | 15.3/16.6 | 15.9/17.5 | 15.4/17.2 |
| Me_2_/Lig_2_ ^2^ | 16.7/21.0 | 15.3/16.3 | 15.2/17.0 | 15.5/17.5 |
| Protein | 24.69 | 19.91 | 20.18 | 20.6 |
| DNA | 27.55 | 22.71 | 22.55 | 23.27 |
| Ligand | 19.88 | 15.38 | 15.64 | 15.79 |
| Water | 31.07 | 25.52 | 26.83 | 26.55 |
| Resolution (Å) | 1.68 | 1.45 | 1.48 | 1.5 |
| No. reflections | 75534 (7463) | 115174 (11274) | 108850 (10719) | 104674 (10370) |
| R_work_/R_free_ | 0.17/0.19 | 0.18/0.19 | 0.17/0.19 | 0.18/0.19 |
| Wilson B | 22.59 | 17.58 | 17.71 | 17.79 |
| Ramachandran | |  |  |  |
| Favored (%) | 99.06 | 99.69 | 99.38 | 100 |
| Outlier (%) | 0 | 0 | 0 | 0 |
| R.m.s. deviations | |  |  |  |
| Bond lengths (Å) | 0.009 | 0.008 | 0.01 | 0.008 |
| Bond angles (˚) | 1.08 | 1.07 | 1.19 | 1.09 |

^1^Data in the highest resolution shell is shown in the parenthesis.

^2^B-factor of metal ions and their protein nucleotide ligands.

|  | 80 s | 160 s | 320 s | 600 s |
| --- | --- | --- | --- | --- |
| **PDB Code** | 8VMS | 8VMT | 8VMU | 8VMV |
| **Data collection** | |  |  |  |
| Wavelength (Å) | 0.9787 | 0.9787 | 0.9787 | 0.9787 |
| Space group | *P3_1_21* | *P3_1_21* | *P3_1_21* | *P3_1_21* |
| Cell dimensions | |  |  |  |
| *a, b, c* (Å) | 113.58 | 113.58 | 113.58 | 113.58 |
|  | 113.58 | 113.58 | 113.58 | 113.58 |
|  | 87.99 | 87.99 | 87.99 | 87.99 |
| *α, β, γ* (˚) | 90, 90, 120 | 90, 90, 120 | 90, 90, 120 | 90, 90, 120 |
| Resolution (Å)^1^ | 37.18 - 1.42 | 42.93 - 1.481 | 37.18 - 1.52 | 37.18 - 1.59 |
|  | (1.471 - 1.42) | (1.534 - 1.481) | (1.574 - 1.52) | (1.574 - 1.52) |
| R_sym_ or R_merge_^1^ | 0.09766 (0.8326) | 0.07917 (0.9958) | 0.08757 (0.8598) | 0.1071 (0.8005) |
| *I*/σ*I*^1^ | 12.89 (1.95) | 17.59 (2.49) | 14.07 (2.11) | 12.43 (2.24) |
| CC^1/2 1^ | 0.998 (0.82) | 0.999 (0.811) | 0.999 (0.851) | 0.998 (0.869) |
| Completeness (%) | 99.89 (99.08) | 99.98 (99.99) | 99.91 (99.37) | 99.97 (100.00) |
| Redundancy^1^ | 10.8 (9.9) | 11.0 (10.6) | 10.9 (10.7) | 11.0 (11.1) |
| No. unique reflections^1^ | 123050 (12056) | 108688 (10781) | 100572 (9925) | 88017 (8714) |
| **Refinement** | |  |  |  |
| Me_1_ | 0.25 Mg^2+^ | 0.5 Mg^2+^ | 0.7 Mg^2+^ | 1.0 Mg^2+^ |
|  | 0.75 Na^+^ | 0.5 Na^+^ | 0.3 Na^+^ |  |
| Me_2_ | 0.2 Mg^2+^ | 0.4 Mg^2+^ | 0.6 Mg^2+^ | 0.9 Mg^2+^ |
|  | 0.8 Na^+^ | 0.6 Na^+^ | 0.4 Na^+^ | 0.1 Na^+^ |
| Product_1_ | 0.25 | 0.5 | 0.7 | 1.0 |
| Product_2_ | 0.2 | 0.4 | 0.6 | 0.9 |
| B-factors |  |  |  |  |
| Me_1_/Lig_1_ ^2^ | 15.4/17.8 | 15.1/18.0 | 15.0/17.6 | 13.8/15.9 |
| Me_2_/Lig_2_ ^2^ | 15.8/17.5 | 15.8/17.5 | 16.3/17.4 | 14.4/15.7 |
| Protein | 20.05 | 20.07 | 20.42 | 19.41 |
| DNA | 22.25 | 22.28 | 23.31 | 22.02 |
| Ligand | 15.54 | 15.38 | 15.93 | 14.57 |
| Water | 26.41 | 26.15 | 26.35 | 24.89 |
| Resolution (Å) | 1.42 | 1.48 | 1.52 | 1.52 |
| No. reflections | 123047 (12056) | 108678 (10781) | 100559 (9925) | 88005 (8714) |
| R_work_/R_free_ | 0.18/0.19 | 0.18/0.20 | 0.18/0.19 | 0.18/0.20 |
| Wilson B | 17.52 | 17.17 | 17.21 | 16.85 |
| Ramachandran | |  |  |  |
| Favored (%) | 99.69 | 99.69 | 99.38 | 99.69 |
| Outlier (%) | 0 | 0 | 0 | 0 |
| R.m.s. deviations | |  |  |  |
| Bond lengths (Å) | 0.008 | 0.009 | 0.009 | 0.022 |
| Bond angles (˚) | 1.06 | 1.1 | 1.12 | 1.55 |

^1^Data in the highest resolution shell is shown in the parenthesis.

^2^B-factor of metal ions and their protein nucleotide ligands.

(**B**) I-PpoI-DNA complex reaction with 500 µM Mg^2+^ at pH 6.0.

| ph6 | PRS (pH 6.0) | 10 s | 20 s |
| --- | --- | --- | --- |
| **PDB Code** | 8VMW | 8VMX | 8VMY |
| **Data collection** | |  |  |
| Wavelength (Å) | 0.9765 | 0.9765 | 0.9765 |
| Space group | *P3_1_21* | *P3_1_21* | *P3_1_21* |
| Cell dimensions | |  |  |
| *a, b, c* (Å) | 113.88 | 113.88 | 113.88 |
|  | 113.88 | 113.88 | 113.88 |
|  | 88.22 | 88.22 | 88.22 |
| *α, β, γ* (˚) | 90, 90, 120 | 90, 90, 120 | 90, 90, 120 |
| Resolution (Å)^1^ | 47.84 - 1.6 | 47.84 - 1.45 | 43.04 - 1.53 |
|  | (1.657 - 1.6) | (1.502 - 1.45) | (1.585 - 1.53) |
| R_sym_ or R_merge_^1^ | 0.06892 (0.8392) | 0.06899 (0.8012) | 0.08801 (0.7433) |
| *I*/σ*I*^1^ | 21.67 (2.41) | 20.39 (2.42) | 14.38 (1.81) |
| CC^1/2 1^ | 0.999 (0.829) | 0.999 (0.826) | 0.999 (0.845) |
| Completeness (%) | 99.88 (99.99) | 99.95 (99.66) | 99.74 (97.54) |
| Redundancy^1^ | 9.9 (9.6) | 9.9 (9.3) | 9.7 (9.2) |
| No. unique reflections^1^ | 86963 (8596) | 116644 (11543) | 99254 (9632) |
| **Refinement** | |  |  |
| Me_1_ | 1.0 Na^+^ | 1.0 Na^+^ | 0.05 Mg^2+^ |
|  |  |  | 0.95 Na^+^ |
| Me_2_ | 1.0 Na^+^ | 1.0 Na^+^ | 1.0 Na^+^ |
| Product_1_ | - | - | 0.05 |
| Product_2_ | - | - | - |
| B-factors |  |  |  |
| Me_1_/Lig_1_ ^2^ | 14.5/16.6 | 13.1/14.7 | 13.0/13.9 |
| Me_2_/Lig_2_ ^2^ | 115.0/15.7 | 13.1/14.2 | 13.2/14.8 |
| Protein | 18.25 | 17.45 | 17.75 |
| DNA | 21.07 | 20.15 | 20.36 |
| Ligand | 14.37 | 13.05 | 13.2 |
| Water | 24.16 | 23.17 | 23.99 |
| Resolution (Å) | 1.6 | 1.45 | 1.53 |
| No. reflections | 86958 (8596) | 116640 (11543) | 99251 (9632) |
| R_work_/R_free_ | 0.18/0.20 | 0.18/0.18 | 0.18/0.19 |
| Wilson B | 15.76 | 15.32 | 15.54 |
| Ramachandran | |  |  |
| Favored (%) | 99.06 | 98.75 | 99.06 |
| Outlier (%) | 0 | 0 | 0 |
| R.m.s. deviations | |  |  |
| Bond lengths (Å) | 0.008 | 0.008 | 0.01 |
| Bond angles (˚) | 1.11 | 1.1 | 1.16 |

^1^Data in the highest resolution shell is shown in the parenthesis.

^2^B-factor of metal ions and their protein nucleotide ligands.

|  | 40 s | 80 s | 160 s |
| --- | --- | --- | --- |
| **PDB Code** | 8VMZ | 8VN0 | 8VN1 |
| **Data collection** | |  |  |
| Wavelength (Å) | 0.9765 | 0.9765 | 0.9765 |
| Space group | *P3_1_21* | *P3_1_21* | *P3_1_21* |
| Cell dimensions | |  |  |
| *a, b, c* (Å) | 113.88 | 113.88 | 113.88 |
|  | 113.88 | 113.88 | 113.88 |
|  | 88.22 | 88.22 | 88.22 |
| *α, β, γ* (˚) | 90, 90, 120 | 90, 90, 120 | 90, 90, 120 |
| Resolution (Å)^1^ | 43.04 - 1.57 | 43.04 - 1.6 | 43.04 - 1.79 |
|  | (1.626 - 1.57) | (1.657 - 1.6) | (1.854 - 1.79) |
| R_sym_ or R_merge_^1^ | 0.06753 (0.8942) | 0.1128 (0.8972) | 0.1035 (0.8058) |
| *I*/σ*I*^1^ | 22.77 (2.47) | 12.65 (2.74) | 16.88 (2.64) |
| CC^1/2 1^ | 1 (0.824) | 0.997 (0.816) | 0.999 (0.849) |
| Completeness (%) | 99.64 (96.64) | 99.96 (99.98) | 99.88 (99.48) |
| Redundancy^1^ | 10.0 (9.6) | 9.9 (9.7) | 9.9 (8.9) |
| No. unique reflections^1^ | 91821 (8875) | 87042 (8595) | 62370 (6119) |
| **Refinement** | |  |  |
| Me_1_ | 0.1 Mg^2+^ | 0.2 Mg^2+^ | 0.3 Mg^2+^ |
|  | 0.9 Na^+^ | 0.8 Na^+^ | 0.7 Na^+^ |
| Me_2_ | 0.1 Mg^2+^ | 0.2 Mg^2+^ | 0.3 Mg^2+^ |
|  | 0.9 Na^+^ | 0.8 Na^+^ | 0.7 Na^+^ |
| Product_1_ | 0.1 | 0.2 | 0.3 |
| Product_2_ | 0.1 | 0.2 | 0.3 |
| B-factors |  |  |  |
| Me_1_/Lig_1_ ^2^ | 12.9/14.8 | 13.0/14.9 | 14.7/16.6 |
| Me_2_/Lig_2_ ^2^ | 13.0/14.5 | 12.9/14.0 | 14.2/15.1 |
| Protein | 17.67 | 17.32 | 17.42 |
| DNA | 19.82 | 19.28 | 20.43 |
| Ligand | 13.12 | 12.76 | 14 |
| Water | 23.98 | 24.15 | 23.84 |
| Resolution (Å) | 1.57 | 1.6 | 1.79 |
| No. reflections | 91816 (8875) | 87034 (8595) | 62365 (6118) |
| R_work_/R_free_ | 0.18/0.20 | 0.18/0.20 | 0.18/0.20 |
| Wilson B | 14.96 | 14.21 | 16.34 |
| Ramachandran | |  |  |
| Favored (%) | 99.38 | 99.38 | 99.38 |
| Outlier (%) | 0 | 0 | 0 |
| R.m.s. deviations | |  |  |
| Bond lengths (Å) | 0.008 | 0.009 | 0.008 |
| Bond angles (˚) | 1.14 | 1.13 | 1.09 |

^1^Data in the highest resolution shell is shown in the parenthesis.

^2^B-factor of metal ions and their protein nucleotide ligands.

|  | 320 s | 600 s | 1200 s |
| --- | --- | --- | --- |
| **PDB Code** | 8VN2 | 8VN3 | 8VN4 |
| **Data collection** | |  |  |
| Wavelength (Å) | 0.9765 | 0.9765 | 0.9765 |
| Space group | *P3_1_21* | *P3_1_21* | *P3_1_21* |
| Cell dimensions | |  |  |
| *a, b, c* (Å) | 113.88 | 113.88 | 113.88 |
|  | 113.88 | 113.88 | 113.88 |
|  | 88.22 | 88.22 | 88.22 |
| *α, β, γ* (˚) | 90, 90, 120 | 90, 90, 120 | 90, 90, 120 |
| Resolution (Å)^1^ | 43.04 - 1.63 | 43.04 - 1.63 | 43.04 - 1.75 |
|  | (1.688 - 1.63) | (1.688 - 1.63) | (1.813 - 1.75) |
| R_sym_ or R_merge_^1^ | 0.09884 (0.8855) | 0.08108 (0.8274) | 0.1042 (0.8633) |
| *I*/σ*I*^1^ | 13.94 (1.90) | 19.75 (2.62) | 13.09 (1.81) |
| CC^1/2 1^ | 0.999 (0.815) | 0.999 (0.844) | 0.998 (0.843) |
| Completeness (%) | 99.87 (99.42) | 99.98 (99.99) | 99.93 (99.73) |
| Redundancy^1^ | 9.8 (9.7) | 10.0 (10.2) | 9.7 (9.8) |
| No. unique reflections^1^ | 82333 (8100) | 82420 (8146) | 66724 (6607) |
| **Refinement** | |  |  |
| Me_1_ | 0.6 Mg^2+^ | 0.7 Mg^2+^ | 0.95 Mg^2+^ |
|  | 0.4 Na^+^ | 0.3 Na^+^ | 0.05 Na^+^ |
| Me_2_ | 0.4 Mg^2+^ | 0.65 Mg^2+^ | 0.95 Mg^2+^ |
|  | 0.6 Na^+^ | 0.35 Na^+^ | 0.05 Na^+^ |
| Product_1_ | 0.6 | 0.65/0.05 | 0.80/0.15 |
| Product_2_ | 0.4 | 0.6/0.05 | 0.70/0.25 |
| B-factors |  |  |  |
| Me_1_/Lig_1_ ^2^ | 14.3/16.0 | 13.9/16.2 | 13.5/15.2 |
| Me_2_/Lig_2_ ^2^ | 14.7/15.0 | 13.9/15.8 | 12.6/15.4 |
| Protein | 17.93 | 17.52 | 18.08 |
| DNA | 20.32 | 20.1 | 20.75 |
| Ligand | 13.85 | 13.49 | 13.19 |
| Water | 25.15 | 24.55 | 24.66 |
| Resolution (Å) | 1.63 | 1.63 | 1.75 |
| No. reflections | 82327 (8099) | 82412 (8145) | 66716 (6607) |
| R_work_/R_free_ | 0.18/0.19 | 0.18/0.19 | 0.18/0.20 |
| Wilson B | 14.97 | 14.9 | 15.67 |
| Ramachandran | |  |  |
| Favored (%) | 99.38 | 99.38 | 99.38 |
| Outlier (%) | 0 | 0 | 0 |
| R.m.s. deviations | |  |  |
| Bond lengths (Å) | 0.008 | 0.009 | 0.009 |
| Bond angles (˚) | 1.17 | 1.16 | 1.13 |

^1^Data in the highest resolution shell is shown in the parenthesis.

^2^B-factor of metal ions and their protein nucleotide ligands.

(**C**) I-PpoI-DNA complex reaction with 500 µM Mg^2+^ at pH 8.0.

|  | PRS (pH 8.0) | 10 s | 20 s |
| --- | --- | --- | --- |
| **PDB Code** | 8VN5 | 8VN6 | 8VN7 |
| **Data collection** | |  |  |
| Wavelength (Å) | 0.97872 | 0.9787 | 0.9787 |
| Space group | *P3_1_21* | *P3_1_21* | *P3_1_21* |
| Cell dimensions | |  |  |
| *a, b, c* (Å) | 113.65 | 113.65 | 113.65 |
|  | 113.65 | 113.65 | 113.65 |
|  | 88.31 | 88.31 | 88.31 |
| *α, β, γ* (˚) | 90, 90, 120 | 90, 90, 120 | 90, 90, 120 |
| Resolution (Å)^1^ | 47.79 - 1.653 | 42.99 - 1.541 | 42.99 - 1.67 |
|  | (1.712 - 1.653) | (1.596 - 1.541) | (1.73 - 1.67) |
| R_sym_ or R_merge_^1^ | 0.0808 (1.057) | 0.07081 (0.9637) | 0.08404 (1.07) |
| *I*/σ*I*^1^ | 17.76 (2.20) | 20.06 (2.26) | 16.93 (2.14) |
| CC^1/2 1^ | 0.999 (0.822) | 0.999 (0.806) | 0.999 (0.811) |
| Completeness (%) | 99.69 (97.12) | 99.70 (97.14) | 99.58 (95.98) |
| Redundancy^1^ | 11.1 (10.8) | 11.0 (10.8) | 11.1 (10.6) |
| No. unique reflections^1^ | 78663 (7600) | 96789 (9311) | 76126 (7246) |
| **Refinement** | |  |  |
| Me_1_ | 1.0 Na^+^ | 0.15 Mg^2+^ | 0.35 Mg^2+^ |
|  |  | 0.85 Na^+^ | 0.65 Na^+^ |
| Me_2_ | 1.0 Na^+^ | 0.15 Mg^2+^ | 0.25 Mg^2+^ |
|  |  | 0.85 Na^+^ | 0.75 Na^+^ |
| Product_1_ | - | 0.15 | 0.35 |
| Product_2_ | - | 0.15 | 0.25 |
| B-factors |  |  |  |
| Me_1_/Lig_1_ ^2^ | 17.3/19.3 | 16.7/18.6 | 16.3/18.4 |
| Me_2_/Lig_2_ ^2^ | 17.1/18.4 | 16.9/18.3 | 16.6/18.4 |
| Protein | 21.73 | 21.68 | 21.36 |
| DNA | 24.16 | 23.65 | 23.57 |
| Ligand | 17.09 | 16.87 | 16.49 |
| Water | 28.9 | 28.62 | 28.61 |
| Resolution (Å) | 1.65 | 1.54 | 1.67 |
| No. reflections | 78651 (7597) | 96776 (9311) | 76108 (7241) |
| R_work_/R_free_ | 0.17/0.19 | 0.18/0.20 | 0.18/0.20 |
| Wilson B | 18.36 | 19.47 | 18.36 |
| Ramachandran | |  |  |
| Favored (%) | 99.38 | 99.06 | 99.69 |
| Outlier (%) | 0 | 0.31 | 0 |
| R.m.s. deviations | |  |  |
| Bond lengths (Å) | 0.008 | 0.008 | 0.009 |
| Bond angles (˚) | 1.12 | 1.1 | 1.11 |

^1^Data in the highest resolution shell is shown in the parenthesis.

^2^B-factor of metal ions and their protein nucleotide ligands.

|  | 40 s | 80 s | 160 s |
| --- | --- | --- | --- |
| **PDB Code** | 8VN8 | 8VN9 | 8VNA |
| **Data collection** | |  |  |
| Wavelength (Å) | 0.9787 | 0.9787 | 0.9787 |
| Space group | *P3_1_21* | *P3_1_21* | *P3_1_21* |
| Cell dimensions | |  |  |
| *a, b, c* (Å) | 113.65 | 113.65 | 113.65 |
|  | 113.65 | 113.65 | 113.65 |
|  | 88.31 | 88.31 | 88.31 |
| *α, β, γ* (˚) | 90, 90, 120 | 90, 90, 120 | 90, 90, 120 |
| Resolution (Å)^1^ | 47.79 - 1.6 | 42.99 - 1.69 | 34.87 - 1.543 |
|  | (1.657 - 1.6) | (1.75 - 1.69) | (1.598 - 1.543) |
| R_sym_ or R_merge_^1^ | 0.08194 (1.012) | 0.08947 (0.9124) | 0.08164 (0.9551) |
| *I*/σ*I*^1^ | 17.51 (2.23) | 15.59 (2.16) | 16.71 (2.22) |
| CC^1/2 1^ | 0.999 (0.79) | 0.999 (0.845) | 0.999 (0.818) |
| Completeness (%) | 99.74 (97.60) | 99.72 (97.37) | 99.98 (99.98) |
| Redundancy^1^ | 11.0 (10.8) | 11.0 (10.5) | 11.0 (11.0) |
| No. unique reflections^1^ | 86597 (8362) | 73645 (7158) | 96719 (9584) |
| **Refinement** | |  |  |
| Me_1_ | 0.45 Mg^2+^ | 0.65 Mg^2+^ | 0.95 Mg^2+^ |
|  | 0.55 Na^+^ | 0.35 Na^+^ | 0.05 Na^+^ |
| Me_2_ | 0.3 Mg^2+^ | 0.5 Mg^2+^ | 0.7 Mg^2+^ |
|  | 0.7 Na^+^ | 0.5 Na^+^ | 0.3 Na^+^ |
| Product_1_ | 0.45 | 0.65 | 0.95 |
| Product_2_ | 0.3 | 0.5 | 0.7 |
| B-factors |  |  |  |
| Me_1_/Lig_1_ ^2^ | 16.8/19.4 | 16.7/19.0 | 16.3/18.3 |
| Me_2_/Lig_2_ ^2^ | 17.3/18.5 | 17.7/18.9 | 16.7/18.6 |
| Protein | 22.2 | 22.85 | 22.03 |
| DNA | 24.43 | 25.14 | 24.45 |
| Ligand | 17.18 | 17.61 | 16.84 |
| Water | 29.42 | 29.72 | 29.13 |
| Resolution (Å) | 1.6 | 1.69 | 1.54 |
| No. reflections | 86585 (8362) | 73632 (7154) | 96712 (9584) |
| R_work_/R_free_ | 0.18/0.19 | 0.18/0.19 | 0.18/0.20 |
| Wilson B | 19.09 | 19.87 | 19.48 |
| Ramachandran | |  |  |
| Favored (%) | 99.69 | 100 | 99.69 |
| Outlier (%) | 0 | 0 | 0 |
| R.m.s. deviations | |  |  |
| Bond lengths (Å) | 0.009 | 0.009 | 0.009 |
| Bond angles (˚) | 1.15 | 1.13 | 1.17 |

^1^Data in the highest resolution shell is shown in the parenthesis.

^2^B-factor of metal ions and their protein nucleotide ligands.

|  | 240 s | 320 s | 600 s |
| --- | --- | --- | --- |
| **PDB Code** | 8VNB | 8VNC | 8VND |
| **Data collection** | |  |  |
| Wavelength (Å) | 0.9787 | 0.9787 | 0.9787 |
| Space group | *P3_1_21* | *P3_1_21* | *P3_1_21* |
| Cell dimensions | |  |  |
| *a, b, c* (Å) | 113.65 | 113.65 | 113.65 |
|  | 113.65 | 113.65 | 113.65 |
|  | 88.31 | 88.31 | 88.31 |
| *α, β, γ* (˚) | 90, 90, 120 | 90, 90, 120 | 90, 90, 120 |
| Resolution (Å)^1^ | 42.99 - 1.72 | 47.79 - 1.623 | 47.79 - 1.602 |
|  | (1.781 - 1.72) | (1.681 - 1.623) | (1.659 - 1.602) |
| R_sym_ or R_merge_^1^ | 0.1043 (0.9116) | 0.08664 (0.9454) | 0.09777 (0.9793) |
| *I*/σ*I*^1^ | 14.41 (2.29) | 16.52 (2.41) | 14.55 (2.57) |
| CC^1/2 1^ | 0.999 (0.844) | 0.999 (0.839) | 0.998 (0.827) |
| Completeness (%) | 99.95 (99.68) | 99.98 (100.00) | 99.98 (100.00) |
| Redundancy^1^ | 11.0 (10.9) | 11.0 (11.1) | 11.2 (11.1) |
| No. unique reflections^1^ | 70052 (6923) | 83221 (8204) | 86506 (8558) |
| **Refinement** | |  |  |
| Me_1_ | 1.0 Mg^2+^ | 1.0 Mg^2+^ | 1.0 Mg^2+^ |
| Me_2_ | 1.0 Mg^2+^ | 1.0 Mg^2+^ | 1.0 Mg^2+^ |
| Product_1_ | 1.0 | 1.0 | 1.0 |
| Product_2_ | 1.0 | 1.0 | 1.0 |
| B-factors |  |  |  |
| Me_1_/Lig_1_ ^2^ | 15.3/16.0 | 14.3/15.4 | 14.3/14.9 |
| Me_2_/Lig_2_ ^2^ | 15.2/16.2 | 14.1/15.6 | 13.4/15.2 |
| Protein | 21.5 | 21.23 | 20.45 |
| DNA | 24.62 | 24.06 | 23.37 |
| Ligand | 16.41 | 15.79 | 15.13 |
| Water | 27.43 | 27.6 | 26.89 |
| Resolution (Å) | 1.72 | 1.62 | 1.6 |
| No. reflections | 70046 (6922) | 83212 (8204) | 86496 (8558) |
| R_work_/R_free_ | 0.18/0.20 | 0.18/0.19 | 0.18/0.19 |
| Wilson B | 19.61 | 18.78 | 17.95 |
| Ramachandran | |  |  |
| Favored (%) | 100 | 99.69 | 99.69 |
| Outlier (%) | 0 | 0 | 0 |
| R.m.s. deviations | |  |  |
| Bond lengths (Å) | 0.009 | 0.009 | 0.008 |
| Bond angles (˚) | 1.16 | 1.16 | 1.1 |

^1^Data in the highest resolution shell is shown in the parenthesis.

^2^B-factor of metal ions and their protein nucleotide ligands.

(**D**) I-PpoI-DNA complex reaction with 500 µM Mn^2+^ at pH 6.0.

|  | 10 s | 20 s | 40 s | 80 s |
| --- | --- | --- | --- | --- |
| **PDB Code** | 8VNE | 8VNF | 8VNG | 8VNH |
| **Data collection** | |  |  |  |
| Wavelength (Å) | 0.9786 | 0.9786 | 0.9786 | 0.9786 |
| Space group | *P3_1_21* | *P3_1_21* | *P3_1_21* | *P3_1_21* |
| Cell dimensions | |  |  |  |
| *a, b, c* (Å) | 114.06 | 114.06 | 114.06 | 114.06 |
|  | 114.06 | 114.06 | 114.06 | 114.06 |
|  | 88.02 | 88.02 | 88.02 | 88.02 |
| *α, β, γ* (˚) | 90, 90, 120 | 90, 90, 120 | 90, 90, 120 | 90, 90, 120 |
| Resolution (Å)^1^ | 32.93 - 1.57 | 37.33 - 1.5 | 34.37 - 1.6 | 49.39 - 1.76 |
|  | (1.626 - 1.57) | (1.554 - 1.5) | (1.657 - 1.6) | (1.823 - 1.76) |
| R_sym_ or R_merge_^1^ | 0.07871 (0.9045) | 0.08083 (1.097) | 0.07564 (0.9743) | 0.1045 (1.001) |
| *I*/σ*I*^1^ | 18.11 (2.53) | 16.89 (2.09) | 19.55 (2.40) | 13.57 (2.13) |
| CC^1/2 1^ | 0.999 (0.841) | 0.999 (0.821) | 0.999 (0.809) | 0.998 (0.832) |
| Completeness (%) | 99.98 (100.00) | 99.99 (100.00) | 99.97 (100.00) | 99.70 (97.24) |
| Redundancy^1^ | 11.1 (11.1) | 11.0 (11.0) | 11.1 (11.0) | 11.0 (10.9) |
| No. unique reflections^1^ | 92218 (9154) | 105592 (10465) | 87153 (8627) | 65495 (6311) |
| **Refinement** | |  |  |  |
| Me_1_ | 0.05 Mn^2+^ | 0.1 Mn^2+^ | 0.15 Mn^2+^ | 0.2 Mn^2+^ |
|  | 0.95 Na^+^ | 0.9 Na^+^ | 0.85 Na^+^ | 0.8 Na^+^ |
| Me_2_ | 0.05 Mn^2+^ | 0.1 Mn^2+^ | 0.15 Mn^2+^ | 0.2 Mn^2+^ |
|  | 0.95 Na^+^ | 0.9 Na^+^ | 0.85 Na^+^ | 0.8 Na^+^ |
| Product_1_ | 0.05 | 0.1 | 0.15 | 0.2 |
| Product_2_ | 0.05 | 0.1 | 0.15 | 0.2 |
| B-factors |  |  |  |  |
| Me_1_/Lig_1_ ^2^ | 15.6/18.4 | 16.4/18.5 | 16.2/18.7 | 14.3/17.8 |
| Me_2_/Lig_2_ ^2^ | 15.9/17.2 | 16.8/17.7 | 16.3/17.4 | 15.0/16.6 |
| Protein | 20.95 | 21.18 | 21.5 | 21.18 |
| DNA | 23.45 | 23.56 | 23.85 | 23.64 |
| Ligand | 16.09 | 16.72 | 16.52 | 15.39 |
| Water | 27.18 | 27.29 | 28.05 | 27.3 |
| Resolution (Å) | 1.57 | 1.5 | 1.6 | 1.76 |
| No. reflections | 92213 (9154) | 105589 (10465) | 87145 (8627) | 65484 (6311) |
| R_work_/R_free_ | 0.18/0.20 | 0.18/0.20 | 0.18/0.19 | 0.18/0.21 |
| Wilson B | 18.8 | 18.7 | 18.63 | 18.68 |
| Ramachandran | |  |  |  |
| Favored (%) | 99.38 | 99.06 | 99.06 | 99.38 |
| Outlier (%) | 0 | 0 | 0 | 0 |
| R.m.s. deviations | |  |  |  |
| Bond lengths (Å) | 0.01 | 0.01 | 0.009 | 0.009 |
| Bond angles (˚) | 1.17 | 1.23 | 1.14 | 1.13 |

^1^Data in the highest resolution shell is shown in the parenthesis.

^2^B-factor of metal ions and their protein nucleotide ligands.

|  | 120 s | 160 s | 240 s | 320 s |
| --- | --- | --- | --- | --- |
| **PDB Code** | 8VNJ | 8VNK | 8VNL | 8VNM |
| **Data collection** | |  |  |  |
| Wavelength (Å) | 0.9786 | 0.9786 | 0.9786 | 0.9786 |
| Space group | *P3_1_21* | *P3_1_21* | *P3_1_21* | *P3_1_21* |
| Cell dimensions | |  |  |  |
| *a, b, c* (Å) | 114.06 | 114.06 | 114.06 | 114.06 |
|  | 114.06 | 114.06 | 114.06 | 114.06 |
|  | 88.02 | 88.02 | 88.02 | 88.02 |
| *α, β, γ* (˚) | 90, 90, 120 | 90, 90, 120 | 90, 90, 120 | 90, 90, 120 |
| Resolution (Å)^1^ | 37.33 - 1.61 | 34.37 - 1.61 | 34.37 - 1.64 | 34.37 - 1.59 |
|  | (1.668 - 1.61) | (1.668 - 1.61) | (1.699 - 1.64) | (1.647 - 1.59) |
| R_sym_ or R_merge_^1^ | 0.09422 (0.8953) | 0.08262 (1.086) | 0.07715 (0.814) | 0.06716 (0.9081) |
| *I*/σ*I*^1^ | 15.78 (2.26) | 21.04 (2.30) | 17.91 (2.66) | 22.99 (2.63) |
| CC^1/2 1^ | 0.999 (0.812) | 0.999 (0.8) | 0.999 (0.825) | 1 (0.829) |
| Completeness (%) | 99.98 (100.00) | 99.97 (100.00) | 99.30 (99.98) | 99.97 (99.99) |
| Redundancy^1^ | 11.0 (11.1) | 11.1 (11.1) | 9.1 (9.0) | 11.2 (11.1) |
| No. unique reflections^1^ | 85576 (8465) | 85577 (8465) | 80455 (8029) | 88820 (8795) |
| **Refinement** | |  |  |  |
| Me_1_ | 0.25 Mn^2+^ | 0.4 Mn^2+^ | 0.6 Mn^2+^ | 0.8 Mn^2+^ |
|  | 0.75 Na^+^ | 0.6 Na^+^ | 0.4 Na^+^ | 0.2 Na^+^ |
| Me_2_ | 0.25 Mn^2+^ | 0.45 Mn^2+^ | 0.65 Mn^2+^ | 0.8 Mn^2+^ |
|  | 0.75 Na^+^ | 0.55 Na^+^ | 0.35 Na^+^ | 0.2 Na^+^ |
| Product_1_ | 0.25 | 0.4 | 0.6 | 0.8 |
| Product_2_ | 0.25 | 0.45 | 0.65 | 0.8 |
| B-factors |  |  |  |  |
| Me_1_/Lig_1_ ^2^ | 16.8/19.1 | 15.8/18.9 | 15.2/17.0 | 15.6/17.0 |
| Me_2_/Lig_2_ ^2^ | 16.6/17.7 | 15.5/17.9 | 14.4/17.1 | 14.3/16.4 |
| Protein | 21.3 | 21.4 | 20.27 | 21.02 |
| DNA | 24.16 | 24.16 | 23.05 | 23.84 |
| Ligand | 16.75 | 16.34 | 15.37 | 15.48 |
| Water | 27.58 | 27.91 | 26.79 | 27.97 |
| Resolution (Å) | 1.61 | 1.61 | 1.64 | 1.59 |
| No. reflections | 85571 (8465) | 85566 (8465) | 80444 (8028) | 88812 (8794) |
| R_work_/R_free_ | 0.18/0.20 | 0.18/0.19 | 0.18/0.19 | 0.17/0.19 |
| Wilson B | 19.46 | 18.85 | 17.28 | 18.12 |
| Ramachandran | |  |  |  |
| Favored (%) | 99.38 | 99.38 | 100 | 100 |
| Outlier (%) | 0 | 0 | 0 | 0 |
| R.m.s. deviations | |  |  |  |
| Bond lengths (Å) | 0.009 | 0.009 | 0.008 | 0.008 |
| Bond angles (˚) | 1.17 | 1.13 | 1.04 | 1.07 |

^1^Data in the highest resolution shell is shown in the parenthesis.

^2^B-factor of metal ions and their protein nucleotide ligands.

|  | 480 s | 600 s |
| --- | --- | --- |
| **PDB Code** | 8VNN | 8VNO |
| **Data collection** | |  |
| Wavelength (Å) | 0.9786 | 0.9786 |
| Space group | *P3_1_21* | *P3_1_21* |
| Cell dimensions | |  |
| *a, b, c* (Å) | 114.06 | 114.06 |
|  | 114.06 | 114.06 |
|  | 88.02 | 88.02 |
| *α, β, γ* (˚) | 90, 90, 120 | 90, 90, 120 |
| Resolution (Å)^1^ | 37.33 - 1.792 | 43.07 - 1.7 |
|  | (1.856 - 1.792) | (1.761 - 1.7) |
| R_sym_ or R_merge_^1^ | 0.1312 (1.102) | 0.08224 (0.9698) |
| *I*/σ*I*^1^ | 13.34 (2.57) | 19.05 (2.56) |
| CC^1/2 1^ | 0.998 (0.831) | 0.999 (0.849) |
| Completeness (%) | 99.97 (99.98) | 99.97 (99.97) |
| Redundancy^1^ | 11.1 (11.1) | 11.1 (11.2) |
| No. unique reflections^1^ | 62269 (6153) | 72812 (7213) |
| **Refinement** | |  |
| Me_1_ | 0.8 Mn^2+^ | 0.9 Mn^2+^ |
|  | 0.2 Na^+^ | 0.1 Na^+^ |
| Me_2_ | 0.8 Mn^2+^ | 0.9 Mn^2+^ |
|  | 0.2 Na^+^ | 0.1 Na^+^ |
| Product_1_ | 0.8 | 0.9 |
| Product_2_ | 0.8 | 0.9 |
| B-factors |  |  |
| Me_1_/Lig_1_ ^2^ | 16.3/17.1 | 16.2/17.2 |
| Me_2_/Lig_2_ ^2^ | 14.7/16.7 | 15.5/17.2 |
| Protein | 22.06 | 21.53 |
| DNA | 25.03 | 24.79 |
| Ligand | 16.66 | 16.45 |
| Water | 28.18 | 27.81 |
| Resolution (Å) | 1.79 | 1.7 |
| No. reflections | 62260 (6152) | 72799 (7211) |
| R_work_/R_free_ | 0.18/0.19 | 0.18/0.19 |
| Wilson B | 19.72 | 19.27 |
| Ramachandran | |  |
| Favored (%) | 100 | 99.69 |
| Outlier (%) | 0 | 0 |
| R.m.s. deviations | |  |
| Bond lengths (Å) | 0.01 | 0.009 |
| Bond angles (˚) | 1.22 | 1.21 |

^1^Data in the highest resolution shell is shown in the parenthesis.

^2^B-factor of metal ions and their protein nucleotide ligands.

(**E**) Other I-PpoI-DNA complexes.

|  | I-PpoI 0.2 M sodium malonate | His98Ala I-PpoI 1 mM Mn^2+^  1800 s | His98Ala I-PpoI 1 mM Mn^2+^  Imidazole, 15 h | I-PpoI 200 mM Mn^2+^ 600 s |
| --- | --- | --- | --- | --- |
| **PDB Code** | 8VNP | 8VNQ | 8VNR | 8VNS |
| **Data collection** | |  |  |  |
| Wavelength (Å) | 1.1272 | 0.9765 | 0.9765 | 0.9765 |
| Space group | *P3_1_21* | *P3_1_21* | *P3_1_21* | *P3_1_21* |
| Cell dimensions | |  |  |  |
| *a, b, c* (Å) | 113.695 | 114.646 | 114.09 | 117.905 |
|  | 113.695 | 114.646 | 114.09 | 117.905 |
|  | 87.994 | 89.057 | 88.412 | 84.743 |
| *α, β, γ* (˚) | 90, 90, 120 | 90, 90, 120 | 90, 90, 120 | 90, 90, 120 |
| Resolution (Å)^1^ | 34.28 - 1.79 | 43.36 - 1.93 | 43.13 - 1.98 | 43.73 - 2.111 |
|  | (1.854 - 1.79) | (1.999 - 1.93) | (2.051 - 1.98) | (2.186 - 2.111) |
| R_sym_ or R_merge_^1^ | 0.05373 (0.1686) | 0.21 (0.782) | 0.1138 (0.8958) | 0.1301 (0.6187) |
| *I*/σ*I*^1^ | 46.56 (15.80) | 6.69 (1.37) | 11.19 (1.62) | 10.97 (2.08) |
| CC^1/2 1^ | 1 (0.995) | 0.989 (0.848) | 0.998 (0.848) | 0.996 (0.905) |
| Completeness (%) | 99.91 (99.95) | 99.50 (95.26) | 99.74 (98.19) | 99.61 (96.97) |
| Redundancy^1^ | 20.5 (20.0) | 9.8 (8.9) | 9.8 (9.4) | 9.7 (9.5) |
| No. unique reflections^1^ | 62043 (6149) | 50811 (4824) | 46448 (4491) | 39247 (3780) |
| **Refinement** | |  |  |  |
| Me_1_ | 1.0 Na | 1.0 Na | 1.0 Na | 0.7 Mn^2+^ |
|  |  |  |  | 0.3 Na^+^ |
| Me_2_ | 1.0 Na | 1.0 Na | 1.0 Na | 0.8 Mn^2+^ |
|  |  |  |  | 0.2 Na^+^ |
| Product_1_ | - | - | - | 0.7 |
| Product_2_ | - | - | - | 0.8 |
| B-factors |  |  |  |  |
| Me_1_/Lig_1_ ^2^ | 12.8/14.6 | 25.1/24.6 | 35.9/33.1 | 27.3/30.5 |
| Me_2_/Lig_2_ ^2^ | 12.7/13.2 | 22.3/22.3 | 34.6/32.5 | 29.0/29.7 |
| Protein | 16.91 | 29.29 | 36.05 | 36.11 |
| DNA | 20.32 | 32.76 | 39.19 | 42.08 |
| Ligand | 13.7 | 23.81 | 33.27 | 29.82 |
| Water | 22.25 | 32.52 | 39.31 | 33.85 |
| Resolution (Å) | 1.79 | 1.93 | 1.98 | 2.11 |
| No. reflections | 62036 (6149) | 50810 (4824) | 46440 (4491) | 39239 (3780) |
| R_work_/R_free_ | 0.17/0.19 | 0.19/0.21 | 0.19/0.22 | 0.22/0.25 |
| Wilson B | 16.51 | 30.06 | 35.07 | 35.57 |
| Ramachandran | |  |  |  |
| Favored (%) | 99.06 | 99.38 | 99.38 | 99.38 |
| Outlier (%) | 0 | 0 | 0 | 0 |
| R.m.s. deviations | |  |  |  |
| Bond lengths (Å) | 0.007 | 0.01 | 0.009 | 0.01 |
| Bond angles (˚) | 0.96 | 1.13 | 1.08 | 1.14 |

^1^Data in the highest resolution shell is shown in the parenthesis.

^2^B-factor of metal ions and their protein nucleotide ligands.

|  | I-PpoI 500 µM Mg^2+^ 1800 s | I-PpoI 70 mM Tl^+^ 1800 s |
| --- | --- | --- |
| **PDB Code** | 8VNT | 8VNU |
| **Data collection** | |  |
| Wavelength (Å) | 1.1271 | 0.9765 |
| Space group | *P3_1_21* | *P3_1_21* |
| Cell dimensions | |  |
| *a, b, c* (Å) | 114.129 | 113.574 |
|  | 114.129 | 113.574 |
|  | 88.74 | 87.695 |
| *α, β, γ* (˚) | 90, 90, 120 | 90, 90, 120 |
| Resolution (Å)^1^ | 33.02 - 1.62 | 47.67 - 2.2 |
|  | (1.678 - 1.62) | (2.279 - 2.2) |
| R_sym_ or R_merge_^1^ | 0.1009 (1.138) | 0.1265 (0.9511) |
| *I*/σ*I*^1^ | 23.30 (2.78) | 12.59 (2.17) |
| CC^1/2 1^ | 0.999 (0.867) | 0.998 (0.837) |
| Completeness (%) | 99.96 (99.96) | 99.91 (100.00) |
| Redundancy^1^ | 20.2 (20.6) | 9.9 (10.0) |
| No. unique reflections^1^ | 84795 (8426) | 33498 (3304) |
| **Refinement** | |  |
| Me_1_ | 1.0 Mg^2+^ | 0.2 Tl^+^ 0.9 Na^+^ |
| Me_2_ | 1.0 Mg^2+^ | 0.2 Tl^+^ 0.8 Na^+^ |
| Product_1_ | 0.75/0.25 | 0 |
| Product_2_ | 0.60/0.40 | 0 |
| B-factors |  |  |
| Me_1_/Lig_1_ ^2^ | 19.2/21.8 | 80/37.4 |
| Me_2_/Lig_2_ ^2^ | 20.5/22.8 | 73.7/36.0 |
| Protein | 23.32 | 35.3 |
| DNA | 26.33 | 37.63 |
| Ligand | 19.65 | 59.35 |
| Water | 30.34 | 39.55 |
| Resolution (Å) | 1.62 | 2.2 |
| No. reflections | 84783 (8426) | 33486 (3306) |
| R_work_/R_free_ | 0.18/0.20 | 0.18/0.22 |
| Wilson B | 21.49 | 36.08 |
| Ramachandran | |  |
| Favored (%) | 98.75 | 98.75 |
| Outlier (%) | 0 | 0.31 |
| R.m.s. deviations | |  |
| Bond lengths (Å) | 0.008 | 0.009 |
| Bond angles (˚) | 1.06 | 1.04 |

^1^Data in the highest resolution shell is shown in the parenthesis.

^2^B-factor of metal ions and their protein nucleotide ligands.
